# A Solitary Fibrous Tumor of the Eyelid

**DOI:** 10.12669/pjms.35.6.1086

**Published:** 2019

**Authors:** Nausheen Hayat, Muhammad Mohsin Afzal, Vijay Dembra

**Affiliations:** 1 Dr. Nausheen Hayat, FCPS (OPTH), MRCSEd OPTH (UK). Consultant ophthalmologist, Orbit and Oculoplastic Consultant, Jinnah Post Graduate Medical Center, Rafiqui Shaheed Road, Karachi, Pakistan; 2 Dr. Muhammad Mohsin Afzal, FCPS II 2^nd^ Year Trainee, Jinnah Post Graduate Medical Center, Rafiqui Shaheed Road, Karachi, Pakistan; 3 Dr. Vijay Dembra, FCPS. Consultant Ophthalmologist, Jinnah Post Graduate Medical Center, Rafiqui Shaheed Road, Karachi, Pakistan

**Keywords:** Solitary fibrous tumor, CD34, STAT6, Orbit

## Abstract

Solitary fibrous tumor are rare and benign, soft tissue growth of any part of the human body including orbit and eyes. This case report describes a middle age female presented with a slow-growing painless mass in the superior orbital sulcus in the left eye. The lesion was surgically excised revealing the microscopic features characteristic of solitary fibrous tumor, with immunohistochemically reactivity for CD34 and STAT6 stains. Solitary fibrous tumor (SFT) is typically cured with complete surgical excision and CD34 immunohistochemistry proved to be a useful adjunct to the microscopic diagnosis of this type of tumor.

## INTRODUCTION

Solitary fibrous tumor (SFT), a benign mesenchymal fibroblast-like tumor, was first described as a primary mesothelial spindle-cell tumor of the pleura in 1931 by Klemperer and Rabin.^1^ With the advent of immunohistochemistry, a fibroblastic origin, occasionally with myofibroblastic differentiation, was firmly established. Solitary fibrous tumors of the eyelids are mostly benign, but malignant forms of the tumor can occur de novo or transform from a low grade lesion. Solitary fibrous tumors have a tendency to present in the superior aspect of the orbit, but can be found in the eyelids, lacrimal gland and lacrimal gland fossa. As a general rule, SFT shows an indolent and nonaggressive clinical course. It is known that a complete cure can be achieved with gross total resection of SFT.^2^,^3^ Herein, we report a case of SFT of the eyelid, a site of origin that has rarely been described. To the best of our knowledge, this is the first case of Solitary fibrous tumor of the eyelid reported in our country.

## CASE REPORT

**Fig. 1:**
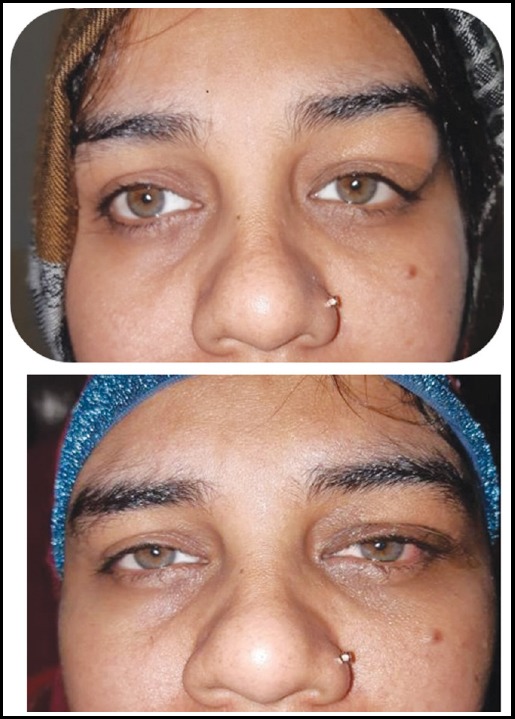
Pre operative, showing left upper lid mass, post operative.

**Fig. 2:**
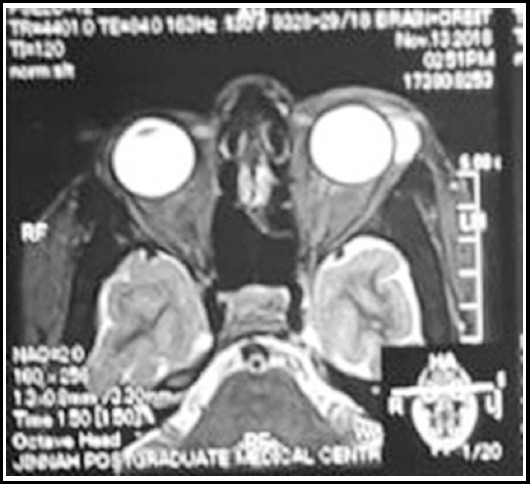
Magnetic resonance imaging (T2) shows hyperintense mass on left lateral aspect.

A 32 years old married female, stay at home mother, reported in orbit oculoplastic clinic of eye ward of our tertiary care center with a chief complain of painless mass with swelling in left lateral portion of upper eyelid for one year (Fig. 1). The mass was small initially, but gradually increased in size causing drooping of upper lid. There was no history of trauma, no significant past history, and no history of any systemic illness. On examination, a soft diffuse mass with ill-defined extent was found on the anterolateral one third portion of left superior sulcus, extending till upper lid margin. The mass was non reducible, transillumination negative and had no pulsation. Overall, the mass caused an S-shaped eye lid deformity. Conjunctival vessels beneath the mass were engorged. Anterior and posterior segment of left eye were unremarkable with no extraocular motility defects. Right eye was normal in examination, whereas BCVA of both eyes was 6/6. Patient was given a trial of oral steroids for a short period by some other physician for which no improvement was documented. When patient reported in our clinic, based on history and clinical examination, Idiopathic orbital inflammatory disease or Pseudotumor was considered. Base line investigations including C-Reactive Protein was carried out, along with magnetic resonance imaging (MRI) were acquired. Laboratory investigations were unremarkable; however, C-REACTIVE PROTEIN was raised accounting 20.66 mg/L (Normal 0.5 – 1.0mg/L). MRI showed a hyperintense well defined mass of about 1.5 ×0.9 cm in left lateral aspect of the eyelid (Fig. 2).

**Fig. 3:**
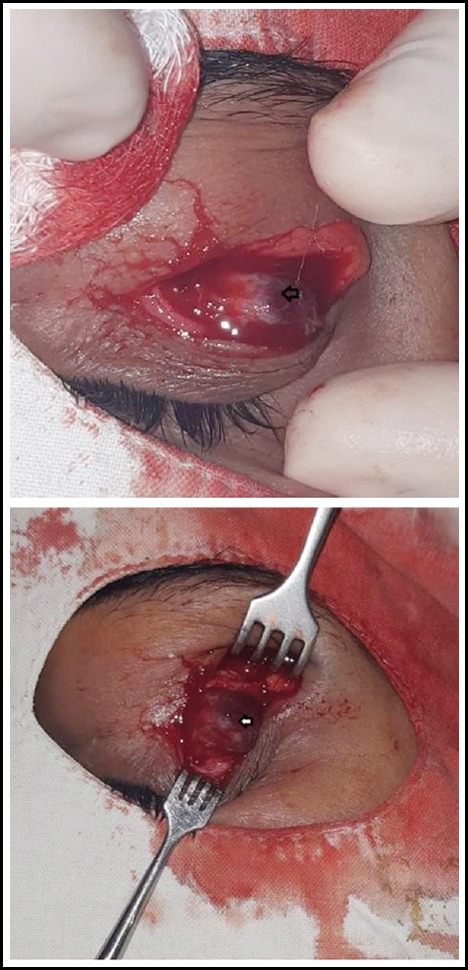
Intraoperative, showing mass (white and black arrow) adherent inferiorly.

We decided to go for an excision biopsy under local anesthesia with all the pre-operative assessments completed. Upon surgical excision, measuring approximately 1.5 x 1.3 x 0.5 cm in maximum diameter a deep reddish brown, nodular tissue with no well-defined margins, adherent to palpebral conjunctiva was identified. However, blood vessels near the mass found moderate to severe engorged and tortuous. Complete excision of the growth carried out and no local invasion or extension was assured (Fig. 3). Subsequently, the defect closed in layers and subcutaneous sutures applied for skin closure. Mass was sent for histopathological examination in one of the largest histopathological center of our city. Post operatively, patient remained well with remarkable improvement in lid drooping and no scarring of incision occurred (Fig. 1).

Microscopic examination revealed spindle cell lesion composed of cells arranged in a storiform pattern with variable areas of congested blood vessels. Individual cells showed scanty cytoplasm with spindle shaped nuclei, whereas scattered multinucleated lesional cells were also present. Moreover, cells showed positivity for CD34 and STAT6 immunohistichemical stains.

## DISCUSSION

SFT is a rare, mostly benign, nevertheless can transform into malignant, and slowly progressive spindle cell tumor originating from mesenchymal fibroblast-like cells.^2^ Age of presentation varies from 20 to 76 years, predominating in middle-aged adults, with no sex predilection. Patients with these tumors typically present with unilateral slowly progressive painless proptosis, eyelid swelling, a palpable mass, tearing, and/or blepharoptosis with occasionally visual disturbances and ocular motility restriction.^4^

With the ongoing research and advanced staining techniques more cases of orbital solitary fibrous tumor are being diagnosed and reported. SFTs can affect any orbital space including lids, lacrimal gland fossa, lacrimal sacs, conjunctivae, and sclera.^5^

On radiological imaging, solitary fibrous tumor usually presents as an isolated soft tissue mass with a smooth, well-circumscribed surface. CT scan will usually show a well-defined soft tissue mass with heterogeneously or homogenously strong enhancement. The MRI findings of SFT describe the mass lesion as isointense or hypointense to gray matter in a T1-weighted image. In a T2-weighted image, the lesion shows hypointensity, hyperintensity, or variegated intensity. When gadolinium is used as contrast, the lesion shows homogeneous enhancement.^6^

The classic histopathological feature of SFT is the presence of spindle cells growing in a haphazard manner in a variable cellular stroma described as “pattern less pattern” or keloid-like hyalinization. Other features are thick bands of collagen interspersed between the tumor cells, alternating hypo-.and hyper-cellular areas, branching thin walled vessels of varying caliber and “staghorn” vascular pattern, similar to Hemangiomapericytoma.^3^

Before the widespread use of immunohistochemical studies and CD34 labelling, the diagnosis of orbital and periorbital SFT based solely on the histopathological findings was confused with other benign lesions, such as fibrous histiocytoma and hemangiopericytoma [HPC]. SFT as an entity is rarely diagnosed clinically.^1^

Immunohistochemical studies show SFTs have strong and diffuse positivity to CD34, vimentin, bcl-2 and STAT6. Among them, SFTs possess strong sensitivity and specificity towards STAT6.^4^ However, they are negative to desmin, cytokeratin, factor VIII-related antigen, S-100, SMA, and muscle-specific actin.^7^ It is necessary to differentiate SFT from hemangioperictyoma, which was difficult in past due to inadequate use of immunohistochemistry.^8^ The later one has an aggressive behavior, high recurrence rate and malignant potential as against the usually benign nature of SFT. Strong and consistent positivity to CD34 is an important diagnostic clue favoring SFT as opposed to hemangiopericytoma which shows inconsistent and weak positivity to CD34.^9^

STAT6 immunohistochemistry was recently established as a reliable method to detect solitary fibrous tumor. Multiple researchers have reported the sensitivity and specificity of STAT6 expression in SFTs and its histopathologic mimickers. In one study the immunoreactivity of 10 SFTs was checked for various markers including CD34 and STAT6. All ten SFTs were CD34 positive, while all other previously performed immunohistochemical stains were unreactive. STAT6 antibody nuclear reactivity was diffusely strong for 9/10 cases, while one case exhibited weak and focal nuclear reactivity.^10^ In our case, the tumor was positive for both CD34 and STAT6.

Eyelid SFT appears to be very rare and so far very few other cases in the HPC-SFT spectrum have been described in the literatures. Previous reports of eyelid lesions from SFT appears to be eyelid extensions or coexisting eyelid symptoms affected by a primary orbital lesion. Most of SFTs behave in a benign fashion, but a local invasion, and recurrences usually follows an incomplete initial excision.

The mainstay of treatment of SFTs is surgical resection with long-term follow-up. In general, SFTs represent a benign disease, and most cases have been treated by local excision. Complete surgical resection is critical to diagnose, treat and prevent recurrence and progression

There have been a very few reported cases of primary SFT of the eyelid, so the information concerning etiologic factors, treatment and prognosis is limited and new additions to the literature may prove useful.

## CONCLUSION

Solitary fibrous tumors are rare tumors of mesenchymal origin for which positive staining for CD34 is the most specific diagnostic test. They generally follow a benign course and their definite treatment is complete surgical excision. In our case the lesion was causing mechanical ptosis and complete surgical excision resulted in good functional and cosmetic outcome.

## Authors’ Contribution:

**NH:** Received the patient, did all examination and performed surgery. Conceived, designed, critical review and final approval.

**MMA:** Assisted in surgery, editing of manuscript, data collection and manuscript writing.

**VD:** Helped in treatment prescription and surgery.
